# An Analysis of Burnout among Female Nurse Educators in Saudi Arabia Using K-Means Clustering

**DOI:** 10.3390/ejihpe13010003

**Published:** 2022-12-30

**Authors:** Nadiah A. Baghdadi, Shatha Khalid Alsayed, Ghalia Amer Malki, Hossam Magdy Balaha, Sally Mohammed Farghaly Abdelaliem

**Affiliations:** 1 Department of Nursing Management and Education, College of Nursing, Princess Nourah Bint Abdulrahman University, P.O. Box 84428, Riyadh 11671, Saudi Arabia; 2 Department of Community Health Nursing, College of Nursing, Princess Nourah Bint Abdulrahman University, P.O. Box 84428, Riyadh 11671, Saudi Arabia; 3 Trinity College Dublin School of Medicine, 152-160 Dublin, Ireland; 4 Computers and Control Systems Engineering Department, Faculty of Engineering, Mansoura University, Mansoura 35516, Egypt; 5 Nursing Administration Department, Faculty of Nursing, Alexandria University, Alexandria 21544, Egypt

**Keywords:** burnout, classification, clustering, deep neural networks (DNN)

## Abstract

Nurse educators are often burnt out and suffer from depression due to their demanding job settings. Biochemical markers of burnout can provide insights into the physiological changes that lead to burnout and may help us prevent burnout symptoms. Research was conducted using a descriptive cross-sectional survey design and a multi-stage sampling method. The ministry of education website provides a list of Saudi Arabian nursing education programs that offer bachelor of science in nursing programs (BSN). The study consisted of 299 qualified participants. Malsach Burnout Inventory (MBI) was used to measure burnout as the dependent variable. The MBI is a 22-item scale that measures depersonalization, accomplishment, and emotional exhaustion during work. Bootstrapping with 5000 replicas was used to address potential non-normality. During this framework, four deep neural networks are created. They all have the same number of layers but differ in the number of neurons they have in the hidden layers. The number of female nurse educators experiencing burnout is moderate (mean = 1.92 ± 0.63). Burnout is also moderately observed in terms of emotional exhaustion (mean = 2.13 ± 0.63), depersonalization (mean = 2.12 ± 0.50), and personal achievement scores (mean = 12 2.38 ± 1.13). It has been shown that stacking the clusters at the end of a column increases their accuracy, which can be considered an important feature when classifying.

## 1. Introduction

Burnout is a psychological concept that refers to experience of emotional exhaustion and depersonalization [1,2]. The global prevalence of burnout among nurse educators has been estimated at 11.23% [3]. Burnout syndrome affects individuals’ psychological and physical statuses and has been the subject of a significant amount of research interest around the world. The increased research on this phenomenon, along with the unfavorable consequences it causes, call for proper estimation of burnout levels. Burnout syndrome is a psychological concept that refers to the worker’s experience of depersonalization and emotional exhaustion. The syndrome affects workers’ physical and psychological status and has been an attractive and significant topic of research around the globe. As it causes unfavorable consequences and results, calls for research on this phenomenon are demanded [1].

The academic culture tends to tolerate faculty performance issues related to burning out. If left unchanged and underestimated, the burnout factors can negatively impact faculty, team, students, teaching, and the program’s quality. Burnout is noticed as job stress and is related to job satisfaction, institutional loyalty, and turnover [4]. Additionally, burnout is defined as a psychological syndrome by Leiter and Maslach (2008) [4] as a delayed response (such as exhaustion, cynicism, and decreased personal accomplishment) to chronic emotional and interpersonal stress at work. It was first used in the service industry. However, as time passed, burnout research gradually expanded to include university students. Following that, the concept of academic burnout was developed. Academic burnout, according to Lian et al. (2014) [5], is a set of negative psychological manifestations in learning (such as anxiety, fatigue, depression, dejection, and low self-esteem) caused by a lack of interest or excessive pressure, which can lead to negative attitudes and behaviors that indicate that the student is tired of learning. Under the stress of long-term learning, students begin to withdraw or refuse to invest in the learning process, which eventually leads to physical or emotional exhaustion, academic inefficacy, and cynicism toward studying. Academic burnout has some negative consequences, including poor academic performance and poor mental health [5]. Consequences of burnout among nurse educators include decreased quality of life, service delivery, and organizational outcomes [2]. This current study focuses on nurses working in academia, who are also prone to experiencing burnout [6,7]. Lackritz [8] estimated the level of burnout among nursing educators at 20%. If left underestimated or unchanged, burnout can negatively impact faculty, team, students, teaching, and the nursing program’s overall quality. Burnout has been linked to decreased job satisfaction and institutional loyalty and increased turnover in educational settings [8]. Additionally, job burnout among nursing educators potentially affects the reputation of nursing colleges, commitment to higher education settings, and the learning experience of nursing students [9]. Understanding the facilitators and barriers of burnout among nursing educators may guide higher education institutions in addressing the shortage of academic staff in nursing colleges [7,10]. Addressing potential sources of burnout is specifically salient during this period of “great resignation”, wherein workers from different sectors, including healthcare and education, tend to easily decide to leave employment because of stressful work conditions and lack of rewards [11].

Meta-analytic evidence suggests that the levels and characteristics of teacher burnout vary across countries and that gender can influence burnout outcomes [12]. Redon-do-Florez et al. [13] demonstrate the significant difference in burnout levels among university professors when grouped according to gender, with females scoring higher compared to their male counterparts. Qualitative evidence demonstrates that, while there is increasing empowerment of women educators in Saudi Arabian society, lack of appreciation and power struggles persist in their work experiences [14]. Given this context, we argue the necessity of conducting a woman-centered study on burnout. Meanwhile, the current educational landscape of Saudi Arabia is influenced by Vision 2030, which stresses the importance of developing attractive, preferred, and stimulating educational environments while connecting these with supportive and integrated services systems. This demand is observed in the objectives of the Saudi Transformation Program, which includes improving recruitment, training, and development of educators. This would then stimulate creativity and an innovative learning environment. This national strategy can potentially impact expectations from educators and, consequently, their demands and burnout. Given these country- and gender-specific contexts, it is necessary and timely to examine burnout and its antecedents, especially in the Middle East region, which is under-represented in nurse burnout research [6].

Identifying the potential reasons for burnout in female nursing colleges will help students as well as educators to understand the correct vision for the workplace objectively. The most common method to assess burnout is self-reported measurements from a psychological standpoint. From the literature review, data mining tools have not yet been utilized commonly to analyze burnout issues. To analyze datasets for obtaining useful knowledge, data mining techniques have been utilized [15]. In the current study, the k-means clustering algorithm is used to split the dataset into k-clusters. Additionally, four deep neural networks are utilized to perform the classification.

### 1.1. Study Contributions

The contributions of the current study can be summarized as follows:

- The k-means clustering algorithm is utilized to cluster the dataset into k-clusters.
- Utilizing four deep neural networks to perform the classification task.
- Reporting state-of-the-art performance metrics and results.

### 1.2. Paper Organization

The subjects discussed by the current study can be summarized as follows: Section 2 discusses the background. It conceptualizes the determinants of burnout. In Section 3, the related studies and literature are reviewed. In Section 4, a discussion occurs regarding the methodology, datasets acquisition, data pre-processing phase, clustering, classification, and performance improvement. Section 5 presents details and discussions of the statistics and experiments. In Section 5 the paper is concluded and future work is presented.

### 1.3. Background

#### Conceptualizing the Determinants of Burnout

For this study, burnout is operationalized as the state of having a high level of emotional exhaustion and depersonalization and a low level of personal achievement in the context of work [2,16]. For the determinants to be examined, this study appeals to the framework by Padilla and Thompson [17], who examined burnout among university doctoral faculty members. In their work, they made use of two theoretical underpinnings. The first is the Job Demand–Control Model [17], which posits that job strain (i.e., burnout) is induced by a mixture of high job demand, low job control, and low social support. The second is the concept of work–family conflict, which suggests that poor job performance and overall wellbeing can result when one’s work disrupts one’s family and personal life and vice versa [17]. From these theories, Padilla and Thompson [17] identified three factors of burnout: task demands, social support, and activities outside work.

Task demands and burnout: Padilla and Thompson [17] operationalized task demands as the hours spent and pressure received by faculty members in performing the following four tasks: teaching, research, service, and grants. This corroborates the assertion of a conceptual review that workloads of faculty members, such as working hours, number of students in class, research productivity, and other career demands, facilitate burnout [14]. Moreover, Zeng et al. [2] suggest that quantitative and qualitative job demands also contribute to occupational burnout among nurses. Additional external demands, such as meeting accreditation and certification requirements, can also compound internal multiple demands and foster burnout among nurse faculty [5]. Empirical evidence among nursing faculty in the northeast US suggests that job demand is positively associated with emotional exhaustion and depersonalization dimensions of burnout [18].

Social support and burnout: For this study, social support refers to the support received from peers, the department, and the college [15]. Zeng et al. [2] consider support from colleagues and the organization as external resources that can protect nurses from occupational burnout. Moreover, Thomas et al. [7] suggest that collegial support through mentors and civil relationships also decreases the risk for burnout. Evidence among Greek nurses suggests that support from friends and significant others is relevant [19]. A narrative review of research among educational professions reveals that lack of social support and participation in decision-making can facilitate burnout among faculty [20].

Activities outside work and burnout: The activities outside work that were considered in this present study are family, leisure, and sleep [21]. These dimensions of life outside work were also noted by Thomas et al. [7], who posited that lack of sleep and exercise and poor work/life balance contributed to nursing faculty burnout. Furthermore, Sabagh et al. [22] suggest that family-related stressors are a determinant of burnout presented in general education literature. Evidence among Canadian nursing faculty linked the ability to accommodate life experiences outside work with decreased emotional exhaustion [23]. Moreover, a study among associate degree nursing program directors noted that sleep problems were linked to burnout, stress, and emotional work demands [24].

Personal and work profile and burnout: Aside from the abovementioned determinants of interest, we also considered contextual factors related to the demographic and work background of the female faculty. Based on a review of education literature, age, years of experience, academic rank, management role, and employment status are gradients that affect the likelihood of faculty burnout [22]. Moreover, because of the increasing population of migrant nursing faculty working in Saudi, we also considered their immigrant status as a potential factor for burnout and other related difficulties, as observed in previous research [25].

According to Maslach (2008) [4], burnout is caused by social interactions between helpers and recipients in which helpers become overly emotionally involved and overextend themselves. This eventually leads to an educational environment. Nurse educators are often in charge of multiple roles and tasks in their organization and work an average of 59 h per week [4]. Time constraints and increasing job demands increase their risk of burnout. Given the link between burnout and physical and emotional health problems [5], identifying stressful and burnout-causing workplace factors in nurse educators is critical. Burnout has serious consequences for nurse educators, students, educational institutions, and, ultimately, the profession. In an era when the profession is facing a global shortage of practicing nurses, highly qualified nurse educators are critical to ensuring that the supply of nurses in the future is adequate to sustain the professional workforce.

### 1.4. Clustering

K-means clustering seeks to split data points into k-clusters in a way that points inside the same cluster are similar and points inside the various clusters are apart [26]. The two points’ similarity is determined by the distance between them. Many methods exist to measure distance. One of the most commonly used distance measurements is Euclidean distance (Minkowski distance with *p* = 2) [27]. The Euclidean distance between two points is calculated using the square of the difference between the x and y coordinates. Clustering offers considerable advantages. It is relatively fast, easy to interpret, scalable for large datasets, and able to select the positions of the initial centroids in a smart way in which convergence is guaranteed and sped up. However, the number of clusters must be pre-determined as it is not possible to guess how many clusters exist in the data. Determining the number of clusters may well be a challenging task. Additionally, only linear boundaries can be conducted. If there is a non-linear structure separating groups in the data, k-means will not be a good choice. It slows down as the number of samples increases. At each step, all data points are accessed by the k-means algorithm and the distances are calculated [28]. An alternative way is to use a subset of data points to update the location of centroids. Additionally, this algorithm is sensitive to outliers.

### 1.5. Classification

A deep neural network is represented as a hierarchical organization of neurons with connections to other neurons. It is a network of neurons with a certain level of complexity (i.e., more than two layers). Deep neural networks use sophisticated mathematical modeling to process data in complex ways. They have an input layer, an output layer, and at least one hidden layer between them. At each layer, specific types of sorting and ordering are performed. The input data are consumed by the neurons in the input layer, which then provide an output to the neurons of the next layer, and the process is repeated up to the last layer, which provides the final output. Each layer consists of one or many neurons, and each computes a small function (i.e., activation function). The activation function simulates the signal to be passed to the next connected neurons. If the input has a value greater than a threshold, the output is passed, or else it is ignored. The connection between two neurons of consecutive layers has a related weight. The weight determines the influence of the input on the output for the subsequent neuron and then for the final output. In a deep neural network, the initial weights are associated with random values. During the training phase, these weights are iteratively updated to conduct better predictions.

### 1.6. K-Fold Cross-Validation

Cross-validation [29] is a technique employed to assess ML models on a limited dataset. Cross-validation is used to detect overfitting. This approach has only one parameter called “K”, with which the input data are split into k-folds. As such, the procedure is often called k-fold cross-validation.

### 1.7. Related Studies

Several researchers have investigated nurse educator burnout. In Alzailai et al. [30], seven databases were explored for published research that examined the factors of job satisfaction and burnout in intensive care unit (ICU) nurses in the Kingdom of Saudi Arabia. Hence, eleven studies related to job satisfaction and burnout and their relating factors were identified. Their study indicated that ICU nurses in Saudi Arabia are suffering from moderate to high levels of burnout. Thomas et al. [7] discussed how to recognize the chronic stress that can lead to burnout, a study for reflection and learning was provided, and strategies to reduce and avoid burnout were offered. Sarmiento et al. [31] employed a descriptive correlational survey to test the model in a sample of 89 Canadian full-time college nurse educators. The educators reported moderate levels of burnout and job satisfaction, as well as moderate levels of empowerment in their workplaces. Shahin et al.’s [32] study aimed to determine the associated factors of burnout among nurses in the primary healthcare centers in the Kingdom of Saudi Arabia. Their study was conducted among 200 nurses by using a self-administered questionnaire. Most participants were females and about 89% scored high at least on one sub-scale of burnout. Al-Omari et al. [1] aimed to evaluate the burnout level and predict the burnout factors of healthcare providers in Saudi Arabia and the United Arab Emirates. A total of 900 healthcare providers were recruited for the study. Their reported results indicated that a high burnout level existed. The participating female healthcare providers had a higher level of emotional exhaustion as compared with their male counterparts. In M. Alqahtani et al. [33], the determinants and magnitude of burnout among emergency physicians and nurses working at hospitals in the cities of Khamis Mushait and Abha were explored. Regarding subjects and methods, the study included 282 physicians and nurses. The majority of the emergency healthcare professionals had high emotional exhaustion. The study showed that a considerable proportion of physicians and nurses working in emergency departments of hospitals in these cities were suffering from particularly high emotional exhaustion, low personal accomplishment, and burnout syndrome. Alqahtani et al.’s [34] study aimed to determine the prevalence and associated risk factors of burnout syndrome among psychiatric nurses in a hospital in Saudi Arabia. In this study, 395 nurses were included through simple random sampling at a psychiatric hospital. Burnout syndrome is prevalent among psychiatric nurses. Alharbi et al.’s [35] study aimed to explore the prevalence of job satisfaction and burnout among Saudi national critical care nurses. A sample of 150 Saudi national critical care nurses from three hospitals in Hail was included in a cross-sectional survey. Saudi national critical care registered nurses reported moderate to high levels of burnout in the areas of emotional exhaustion and dissatisfaction with their jobs.

## 2. Method

**Figure 1:**
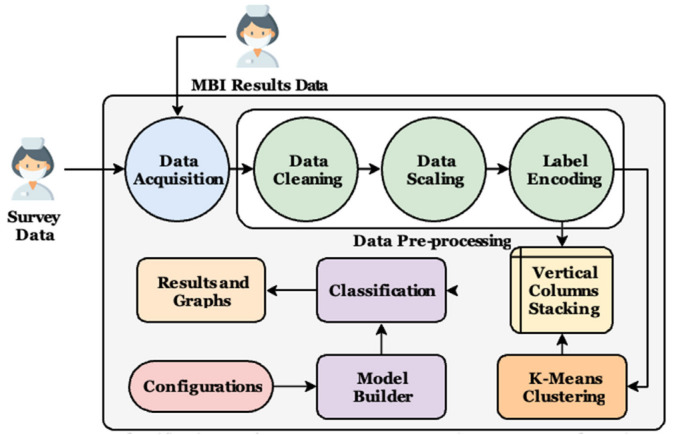
The classification performance improvement using a k-means clustering approach.

The current section presents the details of the suggested approach. In short, it starts by acquiring data manually. After that, a pre-processing phase takes place to make the records more suitable to be processed. The k-means clustering algorithm is used in the clustering phase. Finally, the classification phase is executed, and different performance metrics are reported. The suggested approach is presented in Figure 1.

### 2.1. Data Acquisition

The study used a descriptive, cross-sectional survey research design and a multi-stage sampling design. We obtained a list of nursing education programs in Saudi Arabia that offer a bachelor of science in nursing program (BSN) from the ministry of education website. A number was assigned classification performance improvement using a k-means clustering approach.

Each program and 4 schools/colleges per region were randomly selected using a computerized random number generator. Then, we proceeded to recruit individual faculty members from the selected institutions. The inclusion criteria included female faculty teaching in BSN programs. The exclusion criteria included BSN faculty who are male, managers with no teaching load, and those teaching graduate courses exclusively. Following randomization, faculty names and email addresses from the selected institutions were obtained from the publicly available online directories of the university websites. Based on a priori sample size G-power analysis (version 3.1), the total sample size needed for the hypothesized model is 167 (f2 = 0.15, power = 0.95, α error probability = 0.05). The total number of qualified respondents who participated in the study is 299, with no missed data.

### 2.2. Study Instrumentation

**Personal and work background characteristics:** The first part of the online survey inquired the following details: age, nationality (1 = Saudi local, 0 = non-Saudi), educational attainment (1 = master’s degree, 2 = doctoral degree), academic ranking (1 = teaching assistant and lecturer, 2 = assistant and associate professor), program level assignment (1 = undergraduate only, 2 = with graduate teaching assignment), years in service, and administrative position (1 = yes, 0 = no).

Task demands, social support, and activities outside work: The second section of the online survey measured the task demands, social support, and activities outside work based on the tool used in the previous work of Padilla and Thompson [17]. Task demands have two dimensions: work hours and work pressure. For work hours, the respondents were asked to indicate the number of weekly hours they performed the following tasks: teaching, research, service, and grant writing. For work pressure, the respondents were asked to answer the extent to which they feel pressured to perform the four tasks using a 5-point Likert scale (0 = none, 4 = a very great deal). Social support was measured using three items. The respondents were asked to rate the extent of support they received from three sources (peer, department, and college) using a 5-point Likert scale. For activities outside work, we inquired about the number of hours the respondents spent on sleep, family, and leisure per week. We conducted a pilot study (n = 24) to determine the internal consistency of the tool; the tool yielded an acceptable Cronbach alpha (α = 0.85).

**Malsach Burnout Inventory (MBI):** The dependent variable of the study is burnout and was measured using the Malsach Burnout Inventory (MBI) [4]. MBI is a 22-item scale that measures emotional exhaustion, personal achievement, and depersonalization related to work. A sample item is “Feel working too hard on the job.” Similar to Padilla and Thompson [17], we measured each item using a 5-point Likert scale. Previous studies have estimated the Cronbach alpha of MBI from 0.71 to 0.93. Our pilot study (n = 22) yielded an acceptable score of 0.91.

### 2.3. Data Collection Procedure and Ethical Considerations

Prior to data collection, ethical approval to conduct the study was obtained from Princess Nourah bint Abdulrahman University Institutional Review Board (IRB) (log Number: H-01-R-059). Faculty members from the selected institutions who met the inclusion criteria were invited via email. Before accessing the online survey, a consent statement was provided that included a full explanation of the study and assurance of privacy and confidentiality. The participating faculty were then prompted to click “agree” and complete the online surveys without any incentives. The online survey forms were active from October 2019 to February 2020.

### 2.4. Data Analysis Procedure

Descriptive statistics (i.e., mean and standard deviation for continuous variables, frequency, and percentage for categorical variables) were used to determine the distribution of the variables. Bivariate statistics (i.e., independent *t-*test for dichotomous variables, one-way ANOVA for multinomial variables, and Pearson R correlation for continuous variables) for categorical were used to identify the significant correlates of overall MBI scores. Significant correlates were included in the hierarchical regression model for MBI. The first step included personal and work characteristics, while the second step included task demands, social support, and activities outside work. Bootstrap-237ping using 5000 replicates was used to address possible non-normality. JASP 0.16 was used for analysis. Significance was set at 0.05 level.

### 2.5. Data Pre-Processing

As discussed in the previous section, the authors collected data from the same nurse educators in two different datasets annotated by X1 and X2, where X1 consists of 33 columns while X2 consists of 3 columns. The current phase works on pre-processing the data by applying three cascaded stages. They are (1) data cleaning, (2) data scaling, and (3) label encoding. In the first stage, the data are cleaned by substituting the missing cells with zeros. The second stage performs standardization on the numeric columns using Equation (1), where X is the input image, Xoutput is the scaled image, µ is the image mean, σ is the image standard deviation.

(1) $$X_{output}=\frac{X\;-\;µ}{\sigma }$$

In the third stage, two encoding techniques are used. The first technique is to convert the categorical columns, such as “nationality”, to encoded numeric values. In the current study, the “Saudi” nationality is converted to 1 and 0 otherwise. The second technique is to apply multi-label binarization on the second dataset (i.e., X2). This technique is applied as the same person can have multi-labels, for example, low emotional exhaustion, low depersonalization, and low personal achievement in the MBI data. Each record is converted to a 9-cell binary record. For example, low emotional exhaustion, low depersonalization, and low personal achievement will be converted to [0, 0, 0, 1, 1, 1, 0, 0, 0], where columns are [“HIGH_DP”, “HIGH_EE”, “HIGH_PA”, “LOW_DP”, “LOW_EE”, “LOW_PA”, “MODERATE_DP”, “MODERATE_EE”, “MODERATE_PA”].

#### 2.5.1. Clustering

The k-means clustering algorithm is utilized in the current study to cluster the first dataset (i.e., X1) into k-clusters. The reason behind selecting this clustering approach is that it is computationally very efficient compared to other clustering algorithms. Application of this algorithm can be summarized as follows:

- Pick k-centroids randomly from the data as the initial cluster centers.
- Assign each next data sample to the nearest centroid.
- Move the centroids to the center of the samples that were assigned to them.
- Repeat the second and third steps until the cluster assignments do not change, a specified tolerance is achieved, or a maximum number of iterations is reached.

The squared Euclidean distance (SSD) is used to measure the similarity between different objects. Moreover, the target is to minimize the within-cluster sum of squared errors (SSE) (i.e., cluster inertia) as shown in Equation (2).

(2) $$\mathrm{SSE}=\overset{n}{\underset{i=1}{\sum }}\overset{k}{\underset{j=1}{\sum }}w\left(i,\;j\right)\times {\left|\left|x^{\left(i\right)}-C^{\left(j\right)}\right|\right|}_{2}^{2}$$

where C is the centroid of the j-cluster, w(i, j) is 1 if the x(i) in the j-cluster and 0 otherwise. In addition to the k-means algorithm, the elbow method is used to estimate the optimal number of clusters (i.e., k). Intuitively, if k increases, the within-cluster SSE (i.e., distortion) value should decrease. The idea behind that is to declare the k value where the distortion value begins to decrease rapidly. This phase maps the second dataset (i.e., X2) from a single vertical numeric column that represents the clusters. These columns are added vertically to the first dataset (i.e., X1) at the end. Hence, the number of columns of the modified X1 is 34.

#### 2.5.2. Classification

In the classification phase, four deep neural networks are created. All share the same number of layers but are different in the number of neurons in the hidden layers. The shared structure is built as follows: (1) input layer, (2) hidden layer with a “He Uniform” kernel weights initializer, (3) ReLU activation function, (4) another hidden layer with a “He Uniform” kernel weights initializer, (5) another ReLU activation function, (6) output layer, and (7) output activation function. The number of neurons is summarized in Table 1. The reason behind this is to study the effect of increasing the number of neurons on the classification metrics, as well as to report the lowest model concerning the complexity.

**Figure 2:**
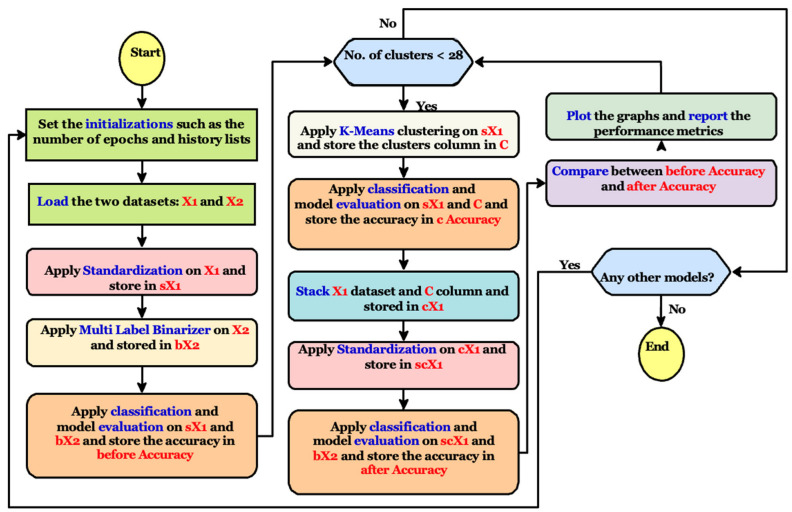
The suggested approach phases and internal processes flowchart summarization.

The classification process is applied two times: the first is performed before adding the clusters columns and the second is applied after adding it. To calculate the clustering accuracy, another classification process is utilized between the first dataset X1 and the clusters column reported from the k-means clustering algorithm. The flowchart presented in Figure 2 shows the phases and internal processes.

#### 2.5.3. Performance Improvement

From Figure 2, the used parameters optimizer is “Adam”. The k-fold cross-validation technique is applied in the current study, where k = 5 folds. In each fold, the accuracy is reported and, finally, the average is taken between the folds as represented in Equation (3). Moreover, the standard deviation is measured between the folds.

(3) $$\mathrm{Average}\;\mathrm{Accuracy}=\frac{1}{K}\times \sum_{i=1}^{K}{\mathrm{Accuracy}}_{i}=\frac{1}{K}\times \sum_{i=1}^{K}\frac{{\mathrm{TP}}_{i}+{\mathrm{TN}}_{i}}{{\mathrm{TP}}_{i}+{\mathrm{TN}}_{i}+{\mathrm{FP}}_{i}+{\mathrm{FN}}_{i}}$$

Where TP, TN, FP, and FN are the true positive, true negative, false positive, and false negative values, respectively. Interventionary studies involving animals or humans, and other studies that require ethical approval, must list the authority that provided approval and the corresponding ethical approval code.

## 3. Results

The current section presents the dataset statistics, framework experiments, and their discussions. Table 2 summarizes the common configurations of all the experiments.

Table 3 shows the descriptive statistical results of the personal and work background characteristics of the participants. A majority of the female nurse educators are within the 41 to 50 years old age bracket (n = 146, 48.8%), non-Saudi (n = 202, 67.6%), with doctoral degree (n = 224, 74.9%), in the assistant to associate professor rank (n = 186, 62.2%), teaching only at the undergraduate level (n = 182, 60.9%), more than 15 years in service (n = 98, 32.8%), and with administrative position (n = 149, 49.8%).

Table 4 shows the mean and standard deviation scores of MBI and its potential predictors. For the task demands, the mean work hours per week is 90.4 ± 13.5, with teaching comprising most of the hours (mean = 23.4 ± 12.9). The overall mean for work pressure is 1.96 ± 0.69, with service pressure as the indicator garnering the highest score (mean = 2.49 ± 1.01). For social support, the overall mean is 1.51 ± 0.89, with college support as the indicator garnering the highest score (mean = 1.57 ± 1.01). Under activities outside work, the average number of hours per day for sleep, family, and leisure are 5.82 ± 1.14, 3.56 ± 2.39, and 2.39 ± 1.75, respectively. Table 4 also indicates that the burnout among the female nurse educators is at a moderate level (mean = 1.92 ± 0.63). As for the specific domains of burnout, the emotional exhaustion (mean = 2.13 ± 0.63), depersonalization (mean = 2.12 ± 0.50), and personal achievement scores (mean = 2.38 ± 1.13) are within a moderate level as well.

Table 5 presents the results of the bivariate tests between the potential predictors and burnout. Among the personal and work characteristics, age (F = 154, *p* < 0.001), nationality (t = −8.11, *p* < 0.001), educational attainment (t = 2.90, *p* = 0.004), years in service (F = 17.4, *p* < 0.001), and administrative position (t = 3.18, *p* = 0.002) were significantly correlated with MBI. Specifically, younger Saudi locals with no doctoral degrees, academic ranking of lecturer or less, lesser years in service, and without administrative positions were observed to have higher burnout scores. Among the two variables under task demands, work pressure (r = 0.346, *p* < 0.0001) was significantly positively correlated with MBI. Social support (r = 0.292, *p* < 0.001) was significantly positively correlated with MBI as well. In terms of activities outside work, sleep (r = −0.219, *p* < 0.001) was significantly negatively correlated with MBI. Work hours per week and family and leisure hours per day did not emerge as significant correlates of burnout.

### 3.1. First Model Experiment

**Figure 3:**
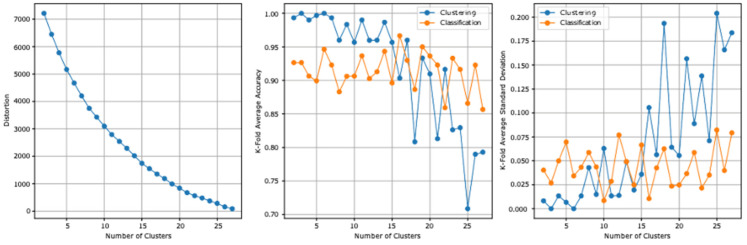
The first model experiment results graphical summarization.

The two datasets report, before the stacking, an accuracy of 59.5% and a standard deviation of 0.126. Table 6 summarizes the first model experiment results after adding the clusters column. From the first experiment reported results, the highest clustering accuracy is 100% and reported at three and six clusters and the lowest standard deviation is 0 at the same clusters. The highest cross-validation classification accuracy is 96.66%, which is reported at 16 clusters, and the lowest standard deviation is 0.008, which is reported at 10 clusters. The lowest distortion is reported at 27 clusters, with a value of 89.013. Figure 3 presents the results graphically.

### 3.2. Second Model Experiment

**Figure 4:**
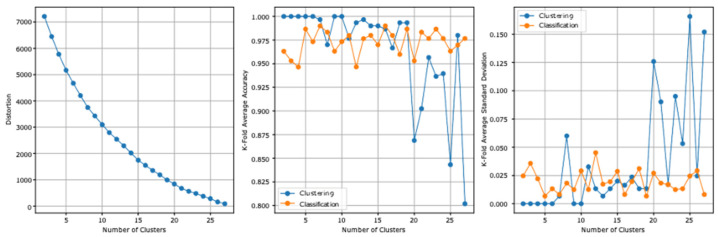
The second model experiment results graphical summarization.

The two datasets report, before the stacking, an accuracy of 82.9% and a standard deviation of 0.047. Table 7 summarizes the second model experiment results after adding the clusters column. From the second experiment reported results, the highest clustering accuracy is 100% and the lowest standard deviation is 0. The highest cross-validation classification accuracy is 99.00%, which is reported at 16 clusters, and the lowest standard deviation is 0.007, which is reported at five clusters. The lowest distortion is reported at 27 clusters, with a value of 89.013. Figure 4 presents the results graphically.

### 3.3. Third Model Experiment

The two datasets report, before the stacking, an accuracy of 94.0% and a standard deviation of 0.029. Table 8 summarizes the third model experiment results after adding the clusters column.

**Figure 5:**
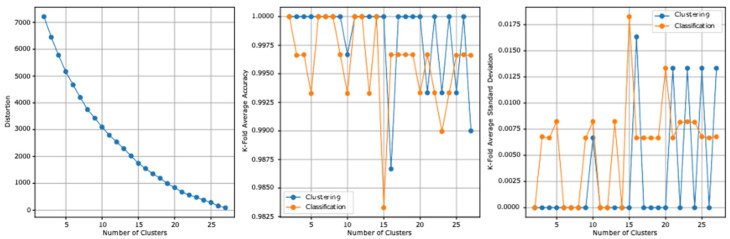
The third model experiment results graphical summarization.

From the third experiment reported results, the highest clustering accuracy is 100% and the lowest standard deviation is 0 at the same clusters. The highest cross-validation classification accuracy is 100% and the lowest standard deviation is 0. The lowest distortion is reported at 27 clusters, with a value of 89.013. Figure 5 presents the results graphically.

### 3.4. Fourth Model Experiment

The two datasets report, before the stacking, an accuracy of 96.6% and a standard deviation of 0.030. Table 9 summarizes the fourth model experiment results after adding the clusters column.

**Figure 6:**
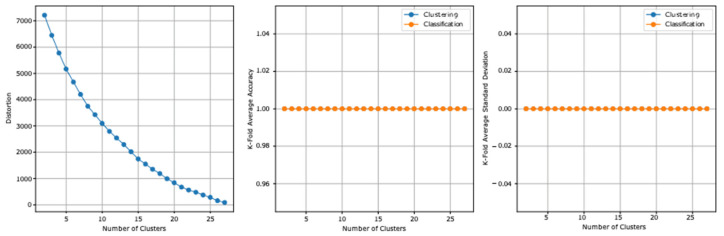
The fourth model experiment results graphical summarization.

From the fourth experiment reported results, the highest clustering accuracy is 100% and reported at all clusters and the lowest standard deviation is 0 at the same clusters. The highest cross-validation classification accuracy is 100%, which is reported at all clusters, and the lowest standard deviation is 0, which is also reported at all clusters. The lowest distortion is reported at 27 clusters, with a value of 89.013. Figure 6 presents the results graphically.

### 3.5. Remarks

From the applied experiments, stacking the clusters at the end of the columns reported higher accuracies, which can be considered as an important feature in the classification process. Table 10 summarizes the best accuracies in the experiments. The first experiment model can be selected concerning the lowest complexity, while the last two experiment models can be selected concerning the reported accuracies.

## 4. Discussion

Burnout-related issues present among faculty are often overlooked by the academic culture. If these factors remain underestimated and unaddressed, they can easily affect the work environment and can negatively affect faculty, staff, students, and teaching quality [8]. Therefore, higher-level institutions need to find and improve aspects of their faculty’s roles that relate to burnout to implement strategies to prevent chronic burnout. The results from the study can be used by higher-level institutions and colleges as guidance to help faculty avoid burnout in support obtaining grants, research activities, peer support, training, and emotional support, among other factors.

It was found that the largest pressures on faculty are experienced in research and service pressures on faculty members. Educators are observed to feel the pressures of research and service more than the pressure of teaching. This could be because faculty members are expected to be at the forefront of their field as it evolves to keep their departments and colleges modern. The constant push and pressure to keep pumping out new information and provide new services highly contributes to the pressures felt by faculty, and these pressures influence burnout rates. Our findings show that, among these pressures, the greatest pressure to conduct research comes from the department and college. Pressure felt by the college is the most frequent. Providing more social support in academia to faculty who are involved in teaching, research, service, and grants application should lessen burnout rates. Their burnout rates should also decrease when there is less pressure to constantly perform. A method to help relieve the research pressure felt by faculty is to support collaboration and collegiality among staff and assure availability of resources to accomplish work. One way to accomplish this is by hiring more support for faculty in the research domain or creating new roles to support faculty in these endeavors. The current study results were consistent with Wang et al. (2021) [36] and Vizoso et al. (2019) [37]. 

The pressure to conduct service was the second most felt pressure by faculty. This is not surprising given that most institutions are research-based; therefore, service is viewed as lesser. Therefore, the finding that service has a high impact on burnout shows that service pressure is more prevalent than institutions may think. The explanation for this is contained in French et al.’s (2020) [38] study, which found that this pressure is experienced by full-time faculty on a research track who are asked to perform in all aspects of the job, unlike part-time faculty who are only asked to teach [38]. These faculty must then take away from their time to conduct research and perform these services, which creates a strain with their priorities. Therefore, a possible solution to this would be to create better social support for service activities by having non-tenured professors help facilitate services, or even creating departmental committees that focus more on such aspects. Another solution could be to train these faculty as part of the hiring process. If they are sufficiently trained and have more experience, these service activities will take less time, making them less straining. Another way to help relieve these pressures is to provide more support for faculty so that they can balance research with their teaching loads. A balance between social support and expectations from job tasks is expected to be a factor that will help to reduce burnout and increase the rate of job satisfaction. This will lead to productive employees. Matching teaching load and student numbers for faculty can reduce the chance of burnout, which can, in turn, reduce teaching pressure. Although this occurs rarely to sometimes, decreasing instances of teaching pressures can contribute to a decreased burnout rate. According to all the statistics, everything interconnects [38,39,40].

The results also show that, on average, faculty experience emotional exhaustion sometimes (2) to frequently (3). This demonstrates a significant implication and contributes greatly to the burnout rate. Based on this, administrators should be aware of the psychological and mental health of the faculty. This also demonstrates evidence that college administrators need to be aware of the factors and negative relationship to emotional exhaustion. Previous research suggested a relationship between gender and burnout due to emotional factors (Van et al., 2022) [39]. Kaiser et al. (2021) [40] revealed that gender differences can significantly affect behaviors related to strain and burnout. Moreover, another study found that female university faculty experience more job and social pressures than their male counterparts and, therefore, had higher burnout rates [40]. These studies provided a basis for the current study and this discussion and are important when discussing these results. Maintaining a healthy work environment also contributes to burnout and quality of work, and the data provided in the study can be used to contribute to help change and make the administration aware of these factors. Overall, this study can be used as a contribution to knowledge of nursing and health sciences programs in terms of faculty management. The findings of the current study must be interpreted considering its limitations. The results rely heavily on subjective perceptions of the participants utilizing self-administered questionnaires rather than clinical diagnosis. It is a cross-sectional study that comprises faculties who work in governmental universities only, which may have impacted the obtained results, making its generalizability limited only to governmental universities.

## 5. Conclusions

Worldwide, there has long been a nursing shortage, and now the pandemic threatens to reduce that number significantly. Nurse educators who work in high-pressure educational environments suffer from psychological stress and burnout. Psychological stress can contribute to burnout, which is characterized by persistent emotional distress. Burnout among nursing faculty is a prominent issue in higher education, and this study shows that there are specific areas that require more support than others. If faculty burnout is a priority among higher education organizations, they should conduct more studies to find the most appropriate solutions, as indicated by the studies. The institutions can also use the information in this study and similar others to help implement better structure and support for faculty. These solutions range from implementation of emotional support for faculty to employing more educators and staff to help support teaching faculty in other parts of their jobs. According to this study, understanding faculty burnout will provide directions for researchers and institutional policies to solve the problems of burnout, shortage, retention, and recruitment of faculty members. Finally, identification of factors that influence burnout could contribute to methods of nursing management to modify job and promotion requirements and help address this issue. From a list of educational programs in Saudi Arabia that offer a bachelor of science in nursing program (BSN) on the ministry of education’s website, an online questionnaire was distributed and 299 qualified participants took part in the study. An analysis of descriptive statistics was conducted to determine the distribution of the variables. Multinomial statistics such as one-way ANOVA and Pearson correlation, as well as categorical statistics, were used to determine the significant correlates of overall MBI scores. Following that, data preprocessing involving cleaning, scaling, and labeling was conducted. As reported in the two datasets, before stacking, they are 59.5% accurate and have a standard deviation of 0.162. With clusters columns added, the accuracy was 100%. The study has limitations in that it is a survey and is, therefore, subject to selection bias. Future studies should examine possible factors affecting nurse educators personally and environmentally. It is important that future research collects data from a diverse group of healthcare educators and workers, including samples from physicians and advanced practice clinicians as well.

Implications of the Study:

This study suggests that strategies to increase work empowerment may help college educators avoid burnout and increase job satisfaction. Nurse educators who are more satisfied with their jobs will feel more joy and accomplishment in their work throughout their academic careers. As a result, student learning will improve by their psychological support and guidance from academic staff and the social workers in the educational environment, and the nursing profession will be more likely to attract highly qualified graduates who will ensure that patients receive the quality of care that they deserve.

Stress, personality, and other personal characteristics, as well as their associations with coping and burnout, could be studied longitudinally in the future. Furthermore, the existence of factors affecting working in relation to stress experience, its nature, and magnitude within the individual necessitates extensive research and analysis using various mixed research methods, in addition to other factors that appear to have a protective effect on the individual and relate to effectiveness in processing emotions, recruiting resources, and managing one’s emotional reactions in the long run. Furthermore, future research may wish to compare different measures and approaches to the concept of burnout and stress, as well as how they affect various stress-related outcomes. Stress reduction techniques can be enhanced through frequently counseling academic staff and students on stress reduction techniques, such as progressive relaxation, meditation, yoga, jogging, and autogenic training. Active practice opportunities may aid in development of these skills. Practicum experiences, comprehensive exams, and other stressful events are common in counselor training programs. Each of these provides an opportunity to coach trainees in becoming more aware of their own stress reactions and to suggest a variety of stress-reduction techniques. Rather than viewing such incidents as something to be avoided at all costs, counselor educators may be able to turn them into teaching opportunities.

## Figures and Tables

**Table 1:** The number of neurons for each used model.

Model	First Hidden	Second Hidden
Model 1	16	16
Model 2	32	16
Model 3	32	32
Model 4	64	32

**Table 2:** The experiment configurations.

Configuration	Specifications
Approaches	Clustering and Classification
Number of Records	299
Apply Dataset Shuffling?	Yes (Random)
Input Image Size	(128 *×* 128 *×* 3)
K-folds	5
Number of Models	4
Number of Epochs	64
Hidden Activation Function	ReLU
Parameters Initializers	He Uniform
Parameters Optimizer	Adam
Elbow Method Range	[2: 27]
Scripting Language	Python
Python Major Packages	Tensorflow, Keras, NumPy, and Matplotlib
Working Environment	Google Colab + GPU

Dataset descriptive and bivariate statistics.

**Table 3:** Personal and work background characteristics (N = 299).

Variables	Categories	n	%
Age	40 years old and below	121	40.5
	41 to 50 years old	146	48.8
	51 years old and above	32	10.7
Nationality	Saudi local	97	32.4
	Non-Saudi	202	67.6
Educational Attainment	Master’s Degree	75	25.1
	Doctoral Degree	224	74.9
Academic Ranking	Teaching Assistant and Lecturer	113	37.8
	Assistant and Associate Professor	186	62.2
Program Level Assignment	Undergraduate only	182	60.9
	With graduate teaching assignment	117	39.1
Years in Service	5 years or less	62	20.7
	6 to 10 years	49	16.4
	10 to 15 years	90	90.1
	More than 15 years	98	32.8
Administrative Position	Yes	150	50.2
	No	149	49.8

**Table 4:** Task demands, social support, activities outside work, and burnout (N = 299).

Variables	Indicators	n	%	Range
Task Demands (Work Hours per Week)	Teaching hours	23.4	12.9	3–60
	Research hours	4.33	4.53	0–20
	Service hours	13.6	12.4	0–45
	Grant hours	2.35	3.31	0–13
	**Overall**	30.4	13.5	6–70
Task Demands (Work Pressure) ^1^	Teaching pressure	1.77	1.02	0–4
	Research pressure	2.11	1.22	0–4
	Service pressure	2.49	1.01	0–4
	Grant pressure	1.48	1.29	0–4
	**Overall**	1.96	0.69	0–4
Social Support ^1^	Peer support	1.49	0.93	0–4
	Departmental support	1.48	0.96	0–4
	College support	1.57	1.01	0–4
	**Overall**	1.51	0.89	0–4
Activities Outside Work (Sleep Hours)	-	5.82	1.14	4–8
Activities Outside Work (Family Hours)	-	3.56	2.39	0–8
Activities Outside Work (Leisure Hours)	-	2.39	1.75	0–8
Burnout (MBI)	Emotional exhaustion	2.13	0.63	0–4
	Depersonalization	2.12	0.5	0–4
	Personal achievement	2.38	1.13	0–4
	**Overall**	1.92	0.61	0–4

Note: ^1^ low = 0.00 to 1.33, moderate = 1.34 to 2.66, and high = 2.67 to 4.00.

**Table 5:** Tests of correlation of personal and work characteristics, task demands, social support, and activities outside work with MBI.

Variables	Categories/Domains	Mean *±* SD	Test Statistic	*p*-Value
Age ^1^	40 years old and below	2.38 ± 0.442	154 ***	<0.001
	41 to 50 years old	1.62 ± 0.541		
	51 years old and above	1.51 ± 0.182		
Nationality ^2^	Saudi local	2.29 ± 0.524	−8.11 ***	<0.001
	Non-Saudi	1.74 ± 0.524		
Educational Attainment ^2^	Master’s Degree	2.09 ± 0.402	2.90 **	0.004
	Doctoral Degree	1.86 ± 0-.658		
Academic Ranking ^2^	Teaching Assistant and Lecturer	2.11 ± 0.446	4.42 **	<0.001
	Assistant and Associate Professor	1.80 ± 0.668		
Program Level Assignment ^2^	Undergraduate only	1.90 ± 0.431	−0.598	0.55
	With graduate teaching assignment	1.94 ± 0.820		
Years in Service ^1^	5 years or less	2.11 ± 0.541	17.4 ***	<0.001
	6 to 10 years	2.21 ± 0.582		
	10 to 15 years	2.00 ± 0.478		
	More than 15 years	1.57 ± 0.627		
Administrative Position ^2^	Yes	1.81 ± 0.636	3.18 **	0.002
	No	2.03 ± 0.568		
Task Demands ^3^	Work hours per week	N/A	−0.027	0.636
	Work pressure	N/A	0.346 ***	<0.001
Social Support ^3^		N/A	0.292 ***	<0.001
Activities outside Work ^3^	Sleep hours per day	N/A	−0.219 ***	<0.001
	Family hours per day	N/A	0.076	0.191
	Leisure hours per day	N/A	−0.069	0.231

Note: ^1^ one-way ANOVA, ^2^ independent *t*-test, ^3^ Pearson R correlation test; ** *p <* 0.01, *** *p <* 0.001.

**Table 6:** The first model experiment results summarization.

Clusters N	Clustering Accuracy	Clustering Std.	Classification Accuracy (After)	Classification Std. (After)	Distortion
2	99.33%	0.008	92.64%	0.040	7212.239
3	100%	0	92.65%	0.027	6447.183
4	99.00%	0.013	90.64%	0.050	5771.351
5	99.67%	0.007	89.93%	0.070	5163.321
6	100%	0	94.64%	0.034	4668.881
7	99.33%	0.013	92.29%	0.043	4197.572
8	95.99%	0.043	88.29%	0.059	3748.422
9	98.33%	0.015	90.62%	0.044	3426.598
10	95.67%	0.063	90.63%	0.008	3095.278
11	98.99%	0.013	93.66%	0.029	2793.872
12	95.98%	0.014	90.25%	0.077	2540.693
13	96.00%	0.049	91.29%	0.049	2289.282
14	98.67%	0.019	94.33%	0.025	2021.041
15	95.67%	0.036	89.62%	0.067	1741.733
16	90.33%	0.106	96.66%	0.011	1546.898
17	95.99%	0.056	92.99%	0.043	1350.480
18	80.84%	0.194	88.64%	0.063	1187.888
19	93.33%	0.064	94.99%	0.023	989.493
20	90.96%	0.056	93.64%	0.025	839.383
21	81.30%	0.157	92.29%	0.037	673.280
22	91.64%	0.089	85.92%	0.059	560.187
23	82.63%	0.139	93.30%	0.022	479.424
24	82.94%	0.071	91.63%	0.035	370.717
25	70.87%	0.204	86.58%	0.082	284.169
26	78.98%	0.166	92.28%	0.040	152.600
27	79.29%	0.184	85.66%	0.079	89.013

**Table 7:** The second model experiment results summarization.

Clusters #	Clustering Accuracy	Clustering Std.	Classification Accuracy (After)	Classification Std. (After)	Distortion
2	100%	0	96.32%	0.025	7212.239
3	100%	0	95.31%	0.036	6447.183
4	100%	0	94.65%	0.022	5771.351
5	100%	0	98.66%	0.007	5163.321
6	100%	0	97.33%	0.013	4668.881
7	99.67%	0.007	98.99%	0.008	4197.572
8	97.00%	0.060	98.33%	0.018	3748.422
9	100%	0	96.32%	0.012	3426.598
10	100%	0	97.33%	0.029	3095.278
11	97.67%	0.033	97.99%	0.013	2793.872
12	99.33%	0.013	94.66%	0.045	2540.693
13	99.67%	0.007	97.65%	0.017	2289.282
14	99.00%	0.013	98.00%	0.019	2021.041
15	99.00%	0.020	96.99%	0.029	1741.733
16	98.67%	0.016	99.00%	0.008	1546.898
17	96.66%	0.024	97.99%	0.020	1350.48
18	99.33%	0.013	95.98%	0.031	1187.888
19	99.33%	0.013	98.66%	0.007	989.493
20	86.88%	0.126	95.31%	0.027	839.383
21	90.24%	0.090	98.33%	0.018	673.28
22	95.66%	0.017	97.66%	0.017	560.187
23	93.66%	0.095	98.66%	0.012	479.424
24	93.95%	0.053	97.66%	0.013	370.717
25	84.32%	0.166	96.33%	0.024	284.169
26	98.00%	0.024	96.97%	0.029	152.6
27	80.19%	0.152	97.66%	0.008	89.013

**Table 8:** The third model experiment results summarization.

Clusters #	Clustering Accuracy	Clustering Std.	Classification Accuracy (After)	Classification Std. (After)	Distortion
2	100%	0	100%	0	7212.239
3	100%	0	99.66%	0.007	6447.183
4	100%	0	99.67%	0.007	5771.351
5	100%	0	99.33%	0.008	5163.321
6	100%	0	100%	0	4668.881
7	100%	0	100%	0	4197.572
8	100%	0	100%	0	3748.422
9	100%	0	99.67%	0.007	3426.598
10	99.67%	0.007	99.33%	0.008	3095.278
11	100%	0	100%	0	2793.872
12	100%	0	100%	0	2540.693
13	100%	0	99.33%	0.008	2289.282
14	100%	0	100%	0	2021.041
15	100%	0	98.33%	0.018	1741.733
16	98.67%	0.016	99.67%	0.007	1546.898
17	100%	0	99.67%	0.007	1350.48
18	100%	0	99.67%	0.007	1187.888
19	100%	0	99.67%	0.007	989.493
20	100%	0	99.33%	0.013	839.383
21	99.33%	0.013	99.67%	0.007	673.28
22	100%	0	99.33%	0.008	560.187
23	99.33%	0.013	98.99%	0.008	479.424
24	100%	0	99.33%	0.008	370.717
25	99.33%	0.013	99.66%	0.007	284.169
26	100%	0	99.67%	0.007	152.6
27	99.00%	0.013	99.66%	0.007	89.013

**Table 9:** The fourth model experiment results summarization.

Clusters #	Clustering Accuracy	Clustering Std.	Classification Accuracy (After)	Classification Std. (After)	Distortion
2	100%	0	100%	0	7212.239
3	100%	0	100%	0	6447.183
4	100%	0	100%	0	5771.351
5	100%	0	100%	0	5163.321
6	100%	0	100%	0	4668.881
7	100%	0	100%	0	4197.572
8	100%	0	100%	0	3748.422
9	100%	0	100%	0	3426.598
10	100%	0	100%	0	3095.278
11	100%	0	100%	0	2793.872
12	100%	0	100%	0	2540.693
13	100%	0	100%	0	2289.282
14	100%	0	100%	0	2021.041
15	100%	0	100%	0	1741.733
16	100%	0	100%	0	1546.898
17	100%	0	100%	0	1350.48
18	100%	0	100%	0	1187.888
19	100%	0	100%	0	989.493
20	100%	0	100%	0	839.383
21	100%	0	100%	0	673.28
22	100%	0	100%	0	560.187
23	100%	0	100%	0	479.424
24	100%	0	100%	0	370.717
25	100%	0	100%	0	284.169
26	100%	0	100%	0	152.6
27	100%	0	100%	0	89.013

**Table 10:** The best accuracies in the experiment’s summarization.

Experiment	Classification Accuracy (Before)	Classification Accuracy (After)	Difference
**First**	59.5%	96.66%	37.16%
**Second**	82.9%	99.00%	16.1%
**Third**	94.0%	100%	6.0%
**Fourth**	96.6%	100%	3.4%

## Data Availability

Not applicable.
